# Di-μ-chlorido-bis­({2-[(4-bromo­phen­yl)­imino­meth­yl]pyridine-κ^2^ *N*,*N*′}­chloridomercury(II))

**DOI:** 10.1107/S1600536809025641

**Published:** 2009-07-11

**Authors:** Ali Mahmoudi, Saeed Dehghanpour, Mehdi Khalaj, Shabnam Pakravan

**Affiliations:** a Department of Chemistry, Islamic Azad University, Karaj Branch, Karaj, Iran; b Department of Chemistry, Alzahra University, PO Box 1993891176, Vanak, Tehran, Iran; c Department of Chemistry, Islamic Azad University, Buinzahra Branch, Qazvin, Iran

## Abstract

The unique Hg^II^ ion in the title centrosymmetric dinuclear complex, [Hg_2_Cl_4_(C_12_H_9_BrN_2_)_2_], is in a distorted trigonal–bipyramidal coordination environment formed by the bis-chelating *N*-heterocyclic ligand, two bridging Cl atoms and one terminal Cl atom. One of the bridging Hg—Cl bonds is significantly longer than the other.

## Related literature

For background information on diimine complexes, see: Dehghanpour & Mahmoudi (2007 ▶); Dehghanpour, Mahmoudi, Khalaj & Salmanpour (2007 ▶). For related crystal structures, see: Mahmoudi *et al.* (2009 ▶); Dehghanpour, Mahmoudi, Khalaj, Salmanpour & Adib (2007 ▶).
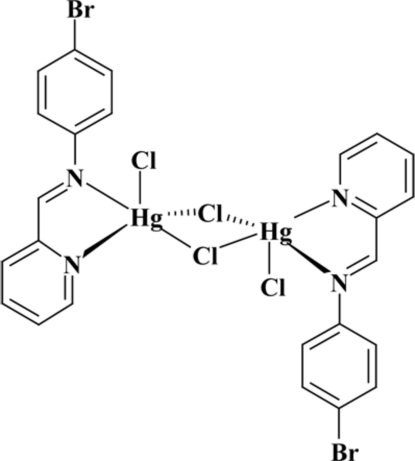


## Experimental

### 

#### Crystal data

[Hg_2_Cl_4_(C_12_H_9_BrN_2_)_2_]
*M* *_r_* = 1065.22
Monoclinic, 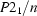

*a* = 7.6697 (2) Å
*b* = 15.0247 (4) Å
*c* = 12.2129 (4) Å
β = 96.738 (1)°
*V* = 1397.63 (7) Å^3^
*Z* = 2
Mo *K*α radiation
μ = 14.24 mm^−1^
*T* = 100 K
0.10 × 0.10 × 0.05 mm

#### Data collection

Bruker SMART APEXII CCD area-detector diffractometer
Absorption correction: multi-scan (*APEX2*; Bruker, 2005 ▶) *T*_min_ = 0.280, *T*_max_ = 0.491
17922 measured reflections
4047 independent reflections
3636 reflections with *I* > 2σ(*I*)
*R*_int_ = 0.032

#### Refinement

*R*[*F* ^2^ > 2σ(*F* ^2^)] = 0.019
*wR*(*F* ^2^) = 0.041
*S* = 1.01
4047 reflections
163 parameters
H-atom parameters constrained
Δρ_max_ = 0.97 e Å^−3^
Δρ_min_ = −1.15 e Å^−3^

### 

Data collection: *APEX2* (Bruker, 2005 ▶); cell refinement: *APEX2*; data reduction: *APEX2*; program(s) used to solve structure: *SHELXTL* (Sheldrick, 2008 ▶); program(s) used to refine structure: *SHELXTL*; molecular graphics: *PLATON* (Spek, 2009 ▶); software used to prepare material for publication: *SHELXTL*.

## Supplementary Material

Crystal structure: contains datablocks global, I. DOI: 10.1107/S1600536809025641/lh2844sup1.cif

Structure factors: contains datablocks I. DOI: 10.1107/S1600536809025641/lh2844Isup2.hkl

Additional supplementary materials:  crystallographic information; 3D view; checkCIF report

## Figures and Tables

**Table:** 

Hg1—N2	2.318 (2)
Hg1—Cl1	2.3799 (7)
Hg1—N1	2.472 (2)
Hg1—Cl2	2.4941 (7)
Hg1—Cl2^i^	2.8799 (6)

**Table:** 

N2—Hg1—Cl1	129.00 (6)
N2—Hg1—N1	70.58 (7)
Cl1—Hg1—N1	107.12 (5)
N2—Hg1—Cl2	102.20 (6)
Cl1—Hg1—Cl2	128.74 (3)
N1—Hg1—Cl2	90.35 (5)
N2—Hg1—Cl2^i^	88.36 (6)
Cl1—Hg1—Cl2^i^	90.07 (2)
N1—Hg1—Cl2^i^	158.28 (5)
Cl2—Hg1—Cl2^i^	88.926 (19)
Hg1—Cl2—Hg1^i^	91.074 (19)

Symmetry code: (i) 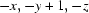.

**Fig. 1.**
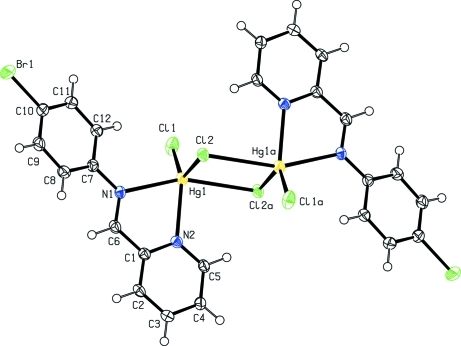
The molecular structure od the title complex, with displacement ellipsoids drawn at the 50% probability level. H atoms are represented as spheres of arbitrary radius [symmetry code: (a) -x, -y+1, -z].

## supplementary crystallographic information

### Comment

In our ongoing studies on the synthesis, structural and spectroscopic characterization of transition metal complexes with diimine ligands (Dehghanpour & Mahmoudi, 2007; Dehghanpour, Mahmoudi, Khalaj, Salmanpour & Adib (2007), we report herein the crystal structure of the title complex.  The title compound was prepared by the reaction of HgCl_2_ with (4-bromophenyl)pyridin-2-ylmethyleneamine.

The molecluar structure of the title complex (I) is shown in (Fig. 1). The unique Hg^II ^ ion in is in a distorted trigonal-bipyramidal coordination environment formed by a bis-chelating ligand, two bridging Cl atoms and one terminal Cl atom. One of the bridging Hg-Cl bonds is significantly longer than the other.

### Experimental

The title complex was prepared by the reaction of HgCl_2_ and (4-bromophenyl)pyridin-2-ylmethyleneamine (molar ratio 1:1) in acetonitrile at room temperature. The solution was then concentrated under vacuum, and diffusion of diethyl ether vapor into the concentrated solution gave yellow crystals of (I) in 69% yield. Calc. for C_12_H_9_BrCl_2_HgN_2_: C 27.06, H 1.70, N 5.26%; found: C 27.01, H 1.72, N 5.20%.

### Refinement

The H atom were placed in calcluated positions with C-H = 0.95Å and refined in a a riding-model approximation with *U*_iso_(H) = 1.2 *U*_eq_(C).

### Crystal data

**Table:**

[Hg_2_Cl_4_(C_12_H_9_BrN_2_)_2_]	*F*(000) = 976
*M**_r_* = 1065.22	*D*_x_ = 2.531 Mg m^−^^3^
Monoclinic, *P*2_1_/*n*	Mo *K*α radiation, λ = 0.71073 Å
Hall symbol: -P 2yn	Cell parameters from 8129 reflections
*a* = 7.6697 (2) Å	θ = 2.2–29.7°
*b* = 15.0247 (4) Å	µ = 14.24 mm^−^^1^
*c* = 12.2129 (4) Å	*T* = 100 K
β = 96.738 (1)°	Prism, yellow
*V* = 1397.63 (7) Å^3^	0.10 × 0.10 × 0.05 mm
*Z* = 2	

### Data collection

**Table:**

Bruker SMART APEXII CCD area-detector diffractometer	4047 independent reflections
Radiation source: fine-focus sealed tube	3636 reflections with *I* > 2σ(*I*)
graphite	*R*_int_ = 0.032
Detector resolution: 0 pixels mm^-1^	θ_max_ = 30.0°, θ_min_ = 2.2°
φ and ω scans	*h* = −10→10
Absorption correction: multi-scan (*APEX2*; Bruker, 2005)	*k* = −21→20
*T*_min_ = 0.280, *T*_max_ = 0.491	*l* = −17→17
17922 measured reflections	

### Refinement

**Table:**

Refinement on *F*^2^	Primary atom site location: structure-invariant direct methods
Least-squares matrix: full	Secondary atom site location: difference Fourier map
*R*[*F*^2^ > 2σ(*F*^2^)] = 0.019	Hydrogen site location: inferred from neighbouring sites
*wR*(*F*^2^) = 0.041	H-atom parameters constrained
*S* = 1.01	*w* = 1/[σ^2^(*F*_o_^2^) + (0.015*P*)^2^ + 1.35*P*] where *P* = (*F*_o_^2^ + 2*F*_c_^2^)/3
4047 reflections	(Δ/σ)_max_ = 0.001
163 parameters	Δρ_max_ = 0.97 e Å^−^^3^
0 restraints	Δρ_min_ = −1.15 e Å^−^^3^

### Special details

**Table:**

Geometry. All e.s.d.'s (except the e.s.d. in the dihedral angle between two l.s. planes) are estimated using the full covariance matrix. The cell e.s.d.'s are taken into account individually in the estimation of e.s.d.'s in distances, angles and torsion angles; correlations between e.s.d.'s in cell parameters are only used when they are defined by crystal symmetry. An approximate (isotropic) treatment of cell e.s.d.'s is used for estimating e.s.d.'s involving l.s. planes.
Refinement. Refinement of *F*^2^ against ALL reflections. The weighted *R*-factor *wR* and goodness of fit *S* are based on *F*^2^, conventional *R*-factors *R* are based on *F*, with *F* set to zero for negative *F*^2^. The threshold expression of *F*^2^ > σ(*F*^2^) is used only for calculating *R*-factors(gt) *etc*. and is not relevant to the choice of reflections for refinement. *R*-factors based on *F*^2^ are statistically about twice as large as those based on *F*, and *R*- factors based on ALL data will be even larger.

### Fractional atomic coordinates and isotropic or equivalent isotropic displacement parameters (Å^2^)

**Table:**

	*x*	*y*	*z*	*U*_iso_*/*U*_eq_	
Hg1	0.170956 (13)	0.446982 (7)	0.108289 (9)	0.02080 (3)	
Br1	0.86964 (5)	0.10383 (2)	−0.01114 (3)	0.04340 (9)	
Cl1	0.42148 (9)	0.53787 (4)	0.15929 (7)	0.03126 (16)	
Cl2	0.04621 (9)	0.39647 (4)	−0.07921 (5)	0.02143 (12)	
N1	0.2551 (3)	0.29234 (14)	0.15931 (18)	0.0183 (4)	
N2	−0.0272 (3)	0.38863 (15)	0.21855 (18)	0.0192 (4)	
C1	−0.0010 (3)	0.30362 (17)	0.2535 (2)	0.0192 (5)	
C2	−0.1169 (4)	0.26078 (18)	0.3146 (2)	0.0207 (5)	
H2A	−0.0974	0.2006	0.3366	0.025*	
C3	−0.2618 (4)	0.30668 (18)	0.3433 (2)	0.0219 (5)	
H3A	−0.3420	0.2788	0.3861	0.026*	
C4	−0.2871 (4)	0.39390 (18)	0.3083 (2)	0.0218 (5)	
H4A	−0.3842	0.4272	0.3275	0.026*	
C5	−0.1679 (4)	0.43198 (18)	0.2445 (2)	0.0209 (5)	
H5A	−0.1878	0.4911	0.2184	0.025*	
C6	0.1522 (4)	0.25556 (18)	0.2214 (2)	0.0212 (5)	
H6A	0.1752	0.1965	0.2471	0.025*	
C7	0.3966 (3)	0.24488 (18)	0.1221 (2)	0.0194 (5)	
C8	0.4078 (4)	0.15213 (19)	0.1228 (2)	0.0239 (5)	
H8A	0.3193	0.1175	0.1508	0.029*	
C9	0.5489 (4)	0.1107 (2)	0.0824 (2)	0.0270 (6)	
H9A	0.5572	0.0476	0.0820	0.032*	
C10	0.6778 (4)	0.1622 (2)	0.0425 (2)	0.0260 (6)	
C11	0.6691 (4)	0.25420 (19)	0.0398 (2)	0.0230 (5)	
H11A	0.7587	0.2884	0.0122	0.028*	
C12	0.5255 (3)	0.29538 (18)	0.0786 (2)	0.0206 (5)	
H12A	0.5151	0.3584	0.0756	0.025*	

### Atomic displacement parameters (Å^2^)

**Table:**

	*U*^11^	*U*^22^	*U*^33^	*U*^12^	*U*^13^	*U*^23^
Hg1	0.02163 (5)	0.01490 (5)	0.02590 (6)	−0.00157 (4)	0.00290 (4)	0.00198 (4)
Br1	0.04489 (19)	0.03626 (17)	0.0548 (2)	0.01823 (15)	0.03004 (17)	0.01083 (16)
Cl1	0.0233 (3)	0.0157 (3)	0.0531 (5)	−0.0016 (2)	−0.0027 (3)	−0.0017 (3)
Cl2	0.0274 (3)	0.0159 (3)	0.0210 (3)	0.0040 (2)	0.0029 (2)	0.0000 (2)
N1	0.0210 (10)	0.0147 (10)	0.0187 (10)	0.0018 (8)	0.0009 (8)	−0.0002 (8)
N2	0.0221 (10)	0.0163 (10)	0.0194 (10)	−0.0008 (8)	0.0028 (8)	0.0005 (8)
C1	0.0227 (12)	0.0171 (12)	0.0179 (12)	0.0002 (9)	0.0025 (10)	0.0003 (9)
C2	0.0278 (13)	0.0152 (12)	0.0189 (12)	−0.0014 (10)	0.0027 (10)	0.0027 (9)
C3	0.0248 (13)	0.0226 (13)	0.0188 (12)	−0.0017 (10)	0.0043 (10)	0.0028 (10)
C4	0.0234 (12)	0.0201 (12)	0.0221 (13)	0.0042 (10)	0.0034 (10)	0.0019 (10)
C5	0.0256 (13)	0.0170 (12)	0.0203 (12)	0.0028 (10)	0.0030 (10)	0.0031 (10)
C6	0.0271 (13)	0.0162 (12)	0.0202 (12)	0.0018 (10)	0.0030 (10)	0.0014 (10)
C7	0.0201 (11)	0.0202 (12)	0.0175 (12)	0.0025 (10)	0.0004 (9)	0.0005 (10)
C8	0.0281 (13)	0.0203 (13)	0.0244 (13)	0.0015 (11)	0.0071 (11)	0.0030 (11)
C9	0.0365 (15)	0.0199 (13)	0.0258 (14)	0.0089 (11)	0.0086 (12)	0.0027 (11)
C10	0.0283 (13)	0.0273 (14)	0.0236 (13)	0.0088 (11)	0.0077 (11)	0.0036 (11)
C11	0.0231 (12)	0.0252 (13)	0.0209 (12)	0.0008 (11)	0.0038 (10)	0.0014 (11)
C12	0.0225 (12)	0.0204 (12)	0.0187 (12)	0.0003 (10)	0.0017 (10)	0.0011 (10)

### Geometric parameters (Å, °)

**Table:**

Hg1—N2	2.318 (2)	C3—H3A	0.9500
Hg1—Cl1	2.3799 (7)	C4—C5	1.392 (4)
Hg1—N1	2.472 (2)	C4—H4A	0.9500
Hg1—Cl2	2.4941 (7)	C5—H5A	0.9500
Hg1—Cl2^i^	2.8799 (6)	C6—H6A	0.9500
Br1—C10	1.894 (3)	C7—C8	1.396 (4)
Cl2—Hg1^i^	2.8799 (6)	C7—C12	1.399 (4)
N1—C6	1.282 (3)	C8—C9	1.389 (4)
N1—C7	1.418 (3)	C8—H8A	0.9500
N2—C5	1.330 (3)	C9—C10	1.388 (4)
N2—C1	1.354 (3)	C9—H9A	0.9500
C1—C2	1.384 (4)	C10—C11	1.385 (4)
C1—C6	1.471 (4)	C11—C12	1.394 (4)
C2—C3	1.387 (4)	C11—H11A	0.9500
C2—H2A	0.9500	C12—H12A	0.9500
C3—C4	1.385 (4)		
			
N2—Hg1—Cl1	129.00 (6)	C3—C4—H4A	120.5
N2—Hg1—N1	70.58 (7)	C5—C4—H4A	120.5
Cl1—Hg1—N1	107.12 (5)	N2—C5—C4	122.4 (2)
N2—Hg1—Cl2	102.20 (6)	N2—C5—H5A	118.8
Cl1—Hg1—Cl2	128.74 (3)	C4—C5—H5A	118.8
N1—Hg1—Cl2	90.35 (5)	N1—C6—C1	120.8 (2)
N2—Hg1—Cl2^i^	88.36 (6)	N1—C6—H6A	119.6
Cl1—Hg1—Cl2^i^	90.07 (2)	C1—C6—H6A	119.6
N1—Hg1—Cl2^i^	158.28 (5)	C8—C7—C12	119.9 (2)
Cl2—Hg1—Cl2^i^	88.926 (19)	C8—C7—N1	123.3 (2)
Hg1—Cl2—Hg1^i^	91.074 (19)	C12—C7—N1	116.8 (2)
C6—N1—C7	121.4 (2)	C9—C8—C7	119.7 (3)
C6—N1—Hg1	113.18 (17)	C9—C8—H8A	120.2
C7—N1—Hg1	125.39 (17)	C7—C8—H8A	120.2
C5—N2—C1	118.8 (2)	C10—C9—C8	119.4 (3)
C5—N2—Hg1	123.96 (18)	C10—C9—H9A	120.3
C1—N2—Hg1	117.19 (17)	C8—C9—H9A	120.3
N2—C1—C2	121.8 (2)	C11—C10—C9	122.1 (3)
N2—C1—C6	118.2 (2)	C11—C10—Br1	119.4 (2)
C2—C1—C6	119.9 (2)	C9—C10—Br1	118.5 (2)
C1—C2—C3	119.3 (2)	C10—C11—C12	118.2 (3)
C1—C2—H2A	120.4	C10—C11—H11A	120.9
C3—C2—H2A	120.4	C12—C11—H11A	120.9
C4—C3—C2	118.6 (2)	C11—C12—C7	120.7 (3)
C4—C3—H3A	120.7	C11—C12—H12A	119.7
C2—C3—H3A	120.7	C7—C12—H12A	119.7
C3—C4—C5	119.0 (2)		
			
N2—Hg1—Cl2—Hg1^i^	88.09 (6)	C6—C1—C2—C3	−179.4 (2)
Cl1—Hg1—Cl2—Hg1^i^	−89.22 (3)	C1—C2—C3—C4	0.9 (4)
N1—Hg1—Cl2—Hg1^i^	158.29 (5)	C2—C3—C4—C5	0.8 (4)
Cl2^i^—Hg1—Cl2—Hg1^i^	0.0	C1—N2—C5—C4	1.5 (4)
N2—Hg1—N1—C6	−0.60 (18)	Hg1—N2—C5—C4	178.0 (2)
Cl1—Hg1—N1—C6	125.53 (18)	C3—C4—C5—N2	−2.1 (4)
Cl2—Hg1—N1—C6	−103.42 (18)	C7—N1—C6—C1	−175.9 (2)
Cl2^i^—Hg1—N1—C6	−15.4 (3)	Hg1—N1—C6—C1	1.6 (3)
N2—Hg1—N1—C7	176.8 (2)	N2—C1—C6—N1	−2.1 (4)
Cl1—Hg1—N1—C7	−57.1 (2)	C2—C1—C6—N1	175.8 (3)
Cl2—Hg1—N1—C7	73.94 (19)	C6—N1—C7—C8	18.2 (4)
Cl2^i^—Hg1—N1—C7	161.92 (14)	Hg1—N1—C7—C8	−159.0 (2)
Cl1—Hg1—N2—C5	86.3 (2)	C6—N1—C7—C12	−164.6 (2)
N1—Hg1—N2—C5	−177.1 (2)	Hg1—N1—C7—C12	18.3 (3)
Cl2—Hg1—N2—C5	−91.0 (2)	C12—C7—C8—C9	1.3 (4)
Cl2^i^—Hg1—N2—C5	−2.5 (2)	N1—C7—C8—C9	178.5 (3)
Cl1—Hg1—N2—C1	−97.14 (19)	C7—C8—C9—C10	0.5 (4)
N1—Hg1—N2—C1	−0.46 (18)	C8—C9—C10—C11	−1.1 (5)
Cl2—Hg1—N2—C1	85.56 (18)	C8—C9—C10—Br1	179.6 (2)
Cl2^i^—Hg1—N2—C1	174.10 (18)	C9—C10—C11—C12	0.0 (4)
C5—N2—C1—C2	0.4 (4)	Br1—C10—C11—C12	179.3 (2)
Hg1—N2—C1—C2	−176.4 (2)	C10—C11—C12—C7	1.8 (4)
C5—N2—C1—C6	178.2 (2)	C8—C7—C12—C11	−2.5 (4)
Hg1—N2—C1—C6	1.4 (3)	N1—C7—C12—C11	−179.8 (2)
N2—C1—C2—C3	−1.6 (4)		

Symmetry codes: (i) −*x*, −*y*+1, −*z*.
